# Expanding HAART Treatment to All Currently Eligible Individuals under the 2008 IAS-USA Guidelines in British Columbia, Canada

**DOI:** 10.1371/journal.pone.0010991

**Published:** 2010-06-07

**Authors:** Viviane D. Lima, Robert S. Hogg, Julio S. G. Montaner

**Affiliations:** 1 British Columbia Centre for Excellence in HIV/AIDS, St. Paul's Hospital, Vancouver, British Columbia, Canada; 2 Division of AIDS, Department of Medicine, Faculty of Medicine, University of British Columbia, Vancouver, British Columbia, Canada; 3 Faculty of Health Sciences, Simon Fraser University, Burnaby, British Columbia, Canada; Tulane University, United States of America

## Abstract

**Background:**

In 2008, the IAS-USA published the revised guidelines for the use of HAART in adults substantially increasing the number of individuals eligible for HAART. The epidemic in British Columbia (BC) is mainly among men who have sex with men and those with injection drug use. Here, we explored the potential impact of different HAART coverage scenarios, based on the new guidelines, on the HIV-related incidence, morbidity and mortality in BC, Canada.

**Methodology:**

We built a mathematical transmission model to investigate different HAART coverage scenarios (50%, 60%, 75% and 100%) of those medically eligible to receive HAART under the 2008 IAS guidelines. All new scenarios were compared to the current coverage in BC under the 2006 IAS guidelines (i.e. baseline scenario). In BC, it is estimated that 25–30% of individuals are unaware of their status. Costs were drug-related and reported in Canadian dollars. HIV-related morbidity and mortality were estimated based on the disability-adjusted life years (DALY) methodology.

**Principal Findings:**

Currently, there are 4379 individuals on HAART under the IAS 2006 guidelines and 6781 individuals who qualify for treatment based on the new guidelines. Within 5 years, increasing HAART coverage decreased yearly new infections by at least 44.8%. In the 50% scenario, in 5 years, DALY decreased by 53% corresponding to 4155 averted DALYs, and in 25 years it decreased by 66% corresponding to 5837 averted DALYs. The effect was even stronger if the 75% scenario was chosen instead. Compared to the 100% expansion scenario, we observed an excess in annual direct treatment expenditures at the end of 5 years of approximately 1 million dollars in the 75% scenario, and of approximately 2 million dollars in the 50% scenario.

**Conclusions/Significance:**

The individual and public health benefits of these new guidelines are immense. The results show that by increasing the number of individuals on HAART save lives, it is cost averting, and it positively impacts society by decreasing the number of new HIV infections. Thus, public health community should consider incremental gains when considering guidelines and policy.

## Introduction

At the end of 2008, after almost three decades since AIDS was first identified, neither a cure nor a fully preventive vaccine is available [1]–[4]. Despite multiple efforts, the epidemic remains an exceptional challenge. It is estimated that, in 2008, 33 million individuals are living with HIV/AIDS in the world, 2.7 million (range 2.2–3.2million) became HIV infected and 2 million (range 1.8–2.3 million) died from AIDS-related causes [5]. Despite substantial prevention efforts and increases in antiretroviral therapy roll-out programs, the epidemic continues its relentless pace. The ability to control HIV/AIDS morbidity and mortality, as well as the spread of HIV has been further compromised as a result of the worldwide financial crisis [6], [7].

It is increasingly apparent that a targeted combination of proven effective interventions will be necessary if we are going to have a chance to control the impact of HIV/AIDS [8]–[12]. Expanding access to antiretroviral treatment to those in medical need has been proposed as a key component of the above mentioned combination prevention approach [8]–[12]. In brief, highly active antiretroviral therapy (HAART) decreases plasma HIV-1-RNA levels to undetectable levels of currently available assays (i.e. <50 copies/mL) in a sustained and reliable fashion. This leads to a dramatic decrease in HIV-1-RNA in sexual secretions (including semen, vaginal fluids and rectal mucosa), which in turn is associated with substantial decrease in the risk of HIV transmission. This effect has now been documented in mother-to-child transmission studies, as well as in observational studies involving discordant heterosexual couples and population-based cohorts [13]–[20]. More recently, further supportive evidence derived from a longitudinal study of injection drug users was presented, where “community” HIV-1-RNA level was found to be the strongest predictor of HIV incidence even after adjusting for traditional risk factors including needle sharing and high-risk sex [19]. Not surprisingly, “community” HIV-1-RNA level in this study was inversely correlated with the use of HAART.

HIV/AIDS related morbidity and mortality have decreased dramatically with the widespread availability of HAART [21]–[23]. Today, HIV/AIDS is seen as a chronic and manageable condition with continued use of HAART, even in resource-limited settings as a result of the relentless roll-out of antiretroviral therapy [24]. Sadly, however, access to HAART remains a challenge throughout the world, even within resource-rich regions [25]–[28]. Egger et al. [25], using data from 42 resource-limited and resource-rich countries (total of 33008 individuals), has shown that in the vast majority of countries the median CD4 cell count at the time of initiation of HAART is substantially below 200 cells/mm^3^. At such a level, the risk of disease progression or death is only partially reversed by HAART [8], [26].

Even in British Columbia (BC), Canada, where there is a universal health care system that provides medical services, including laboratory monitoring, care and antiretroviral therapy free of cost, it has been shown that late or no access to care are major determinants of HIV/AIDS-related mortality [28]. In BC, this tends to be particularly prevalent among rural and urban aboriginal populations, female sex-trade workers, street-involved youth, immigrants from HIV-endemic countries, younger men who have sex with men, as well as among the poor, the homeless and those with mental health and addiction challenges, also referred to as hard-to-reach population [29]–[31]. Here and abroad, the health system has been slow to recognize that facilitated and supported access to care represents a cost-effective means of enhancing HIV/AIDS control, as it relates to HIV/AIDS-related morbidity and mortality as well as HIV transmission.

Recently, the International AIDS Society-USA (IAS-USA) updated the guidelines for the use of HAART in adults infected with HIV [8]. In brief, the 2008 IAS-USA guidelines substantially expanded eligibility to HAART as it recommended that HAART should be offered to all symptomatic HIV infected individuals and to those who are asymptomatic but have CD4 cell counts below 350 cells/mm3. Furthermore, according to the 2008 IAS-USA guidelines, HAART should be offered to all asymptomatic individuals regardless of CD4 cell count if they are at high risk for short-term progression of their disease, as demonstrated by a plasma HIV-1-RNA level above 100,000 copies/mL or an absolute CD4 cell count decrease greater than 100 cells/mm3 within a year. Finally, for the first time based on the new understanding of HIV infection as a chronic inflammatory disease, the 2008 IAS-USA guidelines recommend that HAART should also be offered to all asymptomatic individuals regardless of CD4 cell count if they have an underlying condition or morbidity risk that will be aggravated by untreated HIV infection or that will compromise their future treatment options. Most prevalent among these are history of cardiovascular disease or increased cardiovascular risk, HIV-associated nephropathy, and chronic active hepatitis B or C. Similar recommendations have now been put forward in the USA and in Europe [32]–[33]. Based on these guidelines changes, the number of individuals eligible for HAART has increased substantially. However, no estimates are available regarding the potential impact that the implementation of the 2008 IAS-USA guidelines might have on HIV/AIDS related morbidity and mortality and on HIV transmission in a given population.

Therefore, we undertook the present study to estimate the potential impact of the 2008 IAS-USA guidelines on HIV/AIDS related morbidity and mortality and HIV transmission in BC, via a deterministic mathematical model, in the next 5 (short-term) and 40 (long-term) years, under different HAART expansion scenarios.

We decided to focus on the impact of the new guidelines in BC, given that it houses the British Columbia Centre for Excellence in HIV/AIDS, which has been instrumental in the concept of HAART expansion as a powerful way to curb the HIV epidemic.

## Methods

### Incidence estimates

The BC Centre for Disease Control (BCCDC) produces an annual HIV/AIDS Update report, which includes the number of persons testing newly positive for HIV by year of testing since 1985. Since the year 2000, the number of new positive tests has been relatively stable at approximately 400 cases per year [34], [35]. Additionally, the Public Health Agency of Canada (PHAC) estimates that 30% of those newly infected are not aware of their status, and that HIV incidence in BC is around 340 to 670 new cases per year [36]. Therefore, based on the BCCDC and PHAC estimates, we used the number of positive tests as a proxy for the number of new HIV cases per year.

### Target Population

In BC, at the end of 2005, PHAC estimated that 12300 individuals were infected with HIV [37]. The epidemic affects predominantly men who have sex with men (MSM), followed by injection drug users (IDUs), and heterosexual/non-endemic contacts [36]. Among those infected with HIV, it is estimated that 7380 persons are engaged in care and 4920 are not engaged (i.e. hard-to-reach population [see Introduction for description]). We assumed the same age distribution of those ≥20 years who tested newly positive for HIV in 2006 (N = 393) as reported by the BCCDC, i.e. 94 (24%) in the group 20–29 years, 124 (32%) in the group 30–39 years, 107 (27%) in the group 40–49 years, and 68 (17%) in the 50+ years group [34], [35]. The mortality distribution of our target population in 2006 was obtained from the BC Centre for Excellence in HIV/AIDS Drug Treatment Program, and the overall BC population mortality estimates were obtained from the CANSIM tables from Statistics Canada [38]. To maintain consistency, in all analyses involving age distribution, we used the same age groups as defined in the BCCDC reports. The data in the model consider that between 25% to 30% of individuals are unknow of their HIV status. Also, HIV testing in the province is done solely, free-of-cost, by the BCCDC to all residents in the province, and the numbers increased from 160,554 tests at the end of 2005 to 182,151 tests at the end of 2008, however, for the model purpose we did not include as a separate parameter the number of HIV tests done in the province by BCCDC.The Centre's HIV/AIDS Drug Treatment program has received ethical approval from the University of British Columbia Ethics Review Committee at its St. Paul's Hospital site. The program also conforms with the province's Freedom of Information and Protection of Privacy Act.

### Model Structure

**Figure 1:**
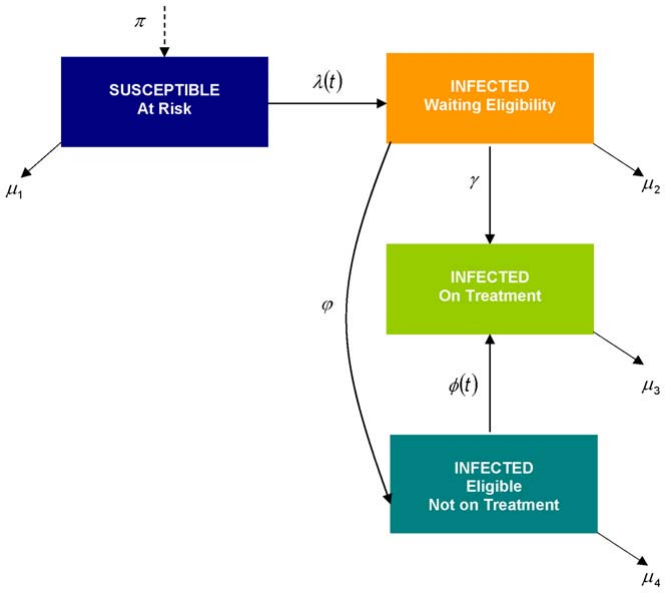
Transmission Model. This diagram shows the distribution of the population at a given point in time. The arrows indicate the movement of individuals across infectivity strata. Parameters' explanation and value are displayed in Table S1.

We built a deterministic transmission model based on the different disease stages of the HIV-1 natural history, whereby high plasma HIV-1-RNA levels being associated with higher chance of disease transmission (Figure 1). The model takes into consideration HIV transmission from those at risk in the main transmission groups in BC (men who have sex with men, injection drug use and female sex workers). We have not included transmissions from heterosexual contact given that this mode of transmission is not the driver of the BC epidemic and that limited data are available to calibrate the necessary parameters associated with this epidemic. The model characterizes the natural history of HIV by three infectivity periods: susceptible; early phase (plasma HIV-1-RNA load <3; ≥3 and <4; ≥4 log_10_ copies/mL); and late stage (CD4 cell count <200 cells/mm^3^). These different transmissions groups were chosen to illustrate that during early phase high viral load is likely to be the main determinant of HIV transmission, and during late phase CD4 cell count plays an additional role in HIV transmission given that in this stage individuals are more likely to develop opportunistic infections. At any of these infectivity periods, we considered death as a possible outcome. The model considers all eligibility criteria for starting therapy as described in the 2008 IAS-USA treatment guidelines [8]. For the analyses presented here, we refer to the IAS-USA guidelines before the year 2008 as the Status Quo scenario. Note that the BC Centre for Excellence is moving away from the Status Quo scenario and adopting the new guidelines. Given the economic crisis and the provincial budget for HIV/AIDS, there was a need to estimate patient and societal benefits based on different HAART expansion scenarios.

The model was updated every month since therapy initiation, and run for 40 years. At each interaction, we generated the number of new infections, the number of individuals eligible for treatment, the number of individuals waiting for treatment and the number of deaths. The model in Figure 1 is specified by four ordinary differential equations:
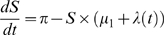


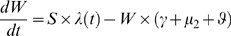


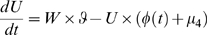


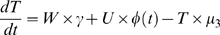
where 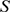: Susceptible and at risk; 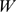: Infected and waiting to meet eligibility to start treatment; 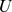: Infected, eligible, but not on treatment; 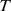: Infected, eligible and on treatment; and *N*, the total population, being defined as 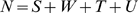. The definitions of the parameters in these four differential equations are presented in Table S1.

As shown in Figure S1, we stratified the HIV infected population in BC based on access to care and eligibility for HAART as: (1) individuals currently receiving HIV treatment and care (N = 4379); (2) individuals who are currently engaged in care, who now meet HAART eligibility criteria under the new guidelines (N = 1602); (3) same as group 2, but despite being well adjusted, decline initiation of treatment (N = 600); (4) individuals who are currently engaged in care, but yet do not meet HAART eligibility criteriae (N = 798); (5) individuals not engaged in care, who should have been on treatment under the previous guidelines because their CD4 cell count is ≤200 cells/mm^3^; (6) and (7) individuals not engaged in care, who now meet the eligibility to start HAART under the new guidelines because their CD4 cell count is ≤350 cells/mm^3^ (N = 1036) or because they meet at least one of the other criteria for therapy initiation (N = 1476), respectively ; and (8) individuals who are not engaged in care, and do not meet the HAART eligibility criteria (N = 342) .We investigated four scenarios of HAART coverage: 50%, 60%, 75% and 100% of all individuals in groups 2, 3, 5, 6 and 7.

The model assumptions were calibrated and validated using the number of individuals testing newly positive for HIV obtained from the BCCDC, and the number of patients enrolled in the Centre's Drug Treatment Program from January of 2006 until the end June of 2008.This model was implemented in Berkeley Madonna Inc. version 8.3.11.

### Cost Estimates

Direct costs were based on treating an individual with first-line therapy in BC during a full year (CAN$ 15800 per year), and no correction was made regarding inflation or discounting (source: Pharmacare, BC Ministry of Health Services). This cost does not include full patient costs or societal costs. An extensive economic evaluation is underway to address both of these previous costs. The lifetime cost was based on a life expectancy of 28.1 years at the age of 20 years in 2006 using the same methodology as in Hogg et al. [22]. Note that different CD4 cell strata might have different life expectancies, but given that the model focused on classifying individuals by different viral load and not CD4 cell count strata, we decided to use the number 28.1 years uniformly across all CD4 cell strata. However, the mortality rates were adjusted for different age groups and model strata.

### Burden of Disease

We used the World Health Organization (WHO) Global Burden of Disease study methodology to assess the impact of disease on the quality of life of the population as a whole [39]. In brief, this approach combines estimates of mortality and morbidity for populations into a single measure of disease burden called disability-adjusted life years (DALY). DALYs are used as a summary measure of population health that reflects the impact of both mortality and non-fatal health decrements. DALYs quantify the gap in years between age at death and some standard age before which death is considered “premature” in addition to time lived in states other than excellent health. DALYs are obtained by summing years of life lost (YLL) from premature death and healthy years lost due to disability (YLD). The key assumptions and decisions made with respect to the estimation of DALY parameters are as follows: a discounting future health rate equal to 3%; age weighting was not utilized; and life long durations were based on the time HIV-positive individuals in BC are expected to live once infected, and calculated using the methodology in Hogg et al. [22] After combining the results for the estimated number of new infections (*I_tj_*) and deaths (*ND_tj_*) for each year *t* (*t* = 1,…,40) and age group *j* (*j* = 20–29, 30–39, 40–49, 50+), the corresponding YLD and YLL estimates for HIV in BC were derived as:
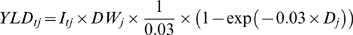


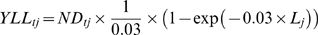
where *DW_j_* represents the disability weight for HIV cases (0.135 for those ≥20 years old^39^); *D_j_* represents the average duration of disability estimated to be 28.1, 25.3, 21.8, 18.2 years for *j* = 20–29, 30–39, 40–49, 50+, respectively; and *L_j_* represents the life expectancy and estimated to be 78.8, 69.3, 59.8, 50.9 years for *j* = 20–29, 30–39, 40–49, 50+, respectively, for the *j^th^* age group. Therefore the DALY estimate, for the *j^th^* age group, and the *t^th^* year, was derived as:
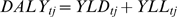



## Results

Using data of 12300 individuals infected with HIV, we were able to estimate the impact of HAART expansion and of the 2008 IAS-USA guidelines on the epidemic in BC. Thus in the short-term (i.e. 5 years), comparing the Status Quo scenario to the 50%, 60%, 75% and 100% HAART expansion based on the 2008 IAS-USA guidelines scenarios, the model estimated that by increasing the number of eligible individuals on treatment by 50%, the total averted infections was 1360 (percent decrease 45%) and in the 100% scenario this number increased to 1498 (percent decrease 49%) averted new infections (Table 1). Table 1 also presents the avoided direct costs associated with each of these scenarios. The 50% scenario generated approximately CAN$ 21.5 million in cost savings, and if the expansion reached a 100% of those in medical need, this figure was increased by approximately CAN$ 2.2 million in just 5 years.

**Table 1:** Effect of HAART expansion on the projected number of new HIV infections and HIV infections averted in the next 5 years, by different scenarios.

IAS-USA Therapeutic Guidelines	HAART expansion scenario	Total number of new infections in 5 years	Averted new infections in 5 years	Percent decrease in new infections in 5 years	5-year Cost Avoidance (Can$)	Lifetime Cost Avoidance (Can$)
2006	None	3,037	0	0	0	0
2008	100%	1,539	1,498	49.3	$23,668,400	$428,398,040
	75%	1,608	1,429	47.1	$22,578,200	$634,447,420
	60%	1,650	1,387	45.7	$21,914,600	$615,800,260
	50%	1,677	1,360	44.8	$21,488,000	$603,812,800

**Figure 2:**
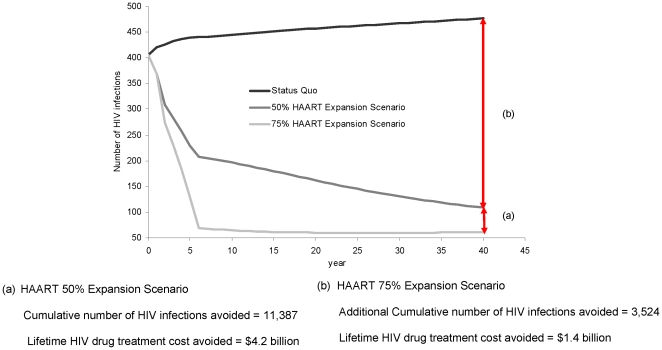
Comparison of the projected number of new HIV infections: Status Quo approach versus 50% and 75% expansion scenarios (over 40 years).

From this point forward, we will compare the 50% and 75% expansion scenarios, as they are practical achievable targets. Figure 2 presents the cumulative number of new infections averted and associated lifetime avoided costs for the 50% and 75% HAART expansion based on the 2008 IAS-USA guidelines scenarios. By enrolling an additional 50% of eligible individuals to HAART, 11387 new HIV infections were averted in 40 years, which is the equivalent of $4.2 billion in avoided HIV lifetime treatment costs that would have been incurred if these individuals had become infected. If this coverage was increased to 75%, we estimated that 14911 new HIV infections were averted in 40 years, which is the equivalent of $5.6 billion in avoided HIV lifetime treatment costs.

The model predicted that in BC, in the next 5 years, if nothing is changed, individuals would loose 6819 years due to premature death (YLL), 1005 healthy years due to disability (YLD) and 7824 DALYs, and the DALYs continue to increase as times passes by, reaching 8891 in 25 years (14% increase) (Table 2). If the HAART expansion based on the 2008 IAS-USA guidelines 50% scenario is achieved, in 5 years DALY decreases by 53% corresponding to 4155 averted DALYs, and in 25 years it decreases by 66% corresponding to 5837 averted DALYs. Table 2 shows the effect is even stronger if the 75% HAART expansion based on the 2008 IAS-USA guidelines scenario is achieved instead.

**Table 2:** Burden of Disease of Status Quo and 50% and 75% HAART expansion scenarios.

Scenario	YLL	YLD	DALY	YLD/YLL	DALYs Averted
Status Quo					
5 years	6,819	1,005	7,824	0.147	
10 years	7,114	1,017	8,132	0.143	
15 years	7,402	1,033	8,435	0.140	
25 years	7,834	1,057	8,891	0.135	
HAART Expansion 50%					
5 years	3,146	523	3,669	0.166	4,155
10 years	3,087	450	3,536	0.146	4,595
15 years	2,982	410	3,393	0.138	5,042
25 years	2,722	332	3,054	0.122	5,837
HAART Expansion 75%					
5 years	1,882	295	2,176	0.157	5,647
10 years	1,779	148	1,926	0.083	6,205
15 years	1,699	140	1,840	0.083	6,595
25 years	1,585	136	1,721	0.086	7,170

YLL: years of life lost; YLD: healthy years lost due to disability; DALY: disability-adjusted life years; DALY averted, for a given year, was calculated in reference to the Status Quo scenario.

**Figure 3:**
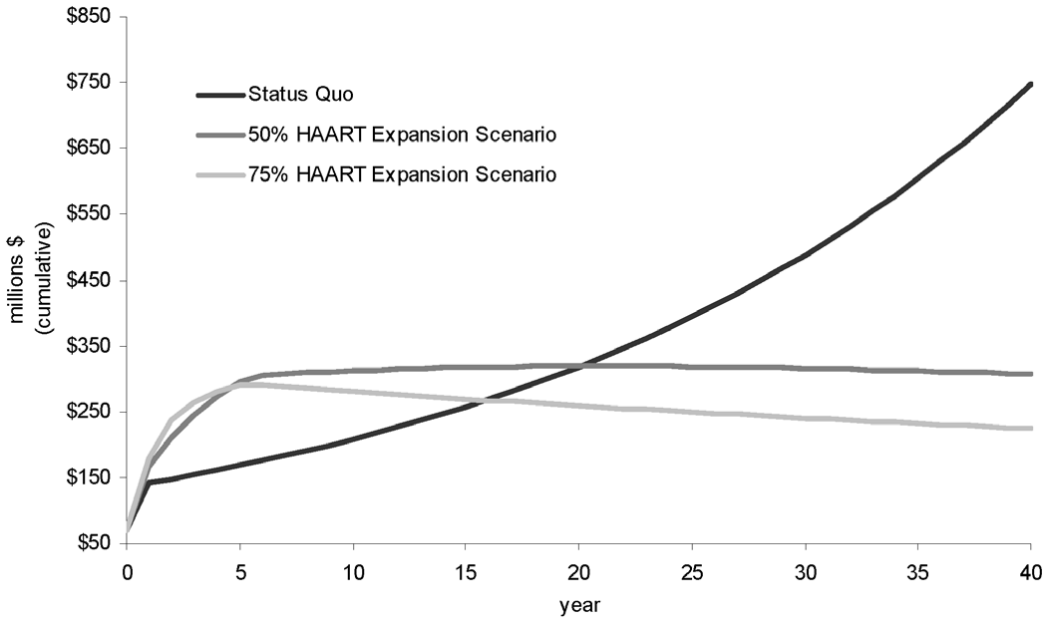
Return on increased investment resulting from implementation of the Status Quo approach versus 50% and 75% expansion scenarios (over 40 years).

Given that HAART expansion based on the 2008 IAS-USA guidelines will require significant investments, it is important to also look at the return on increase investment as a result of the HAART expansion initiative. We achieved this by plotting the projected annual HIV drug treatment cost over 40 years as per the Status Quo, versus the projected annual HIV drug treatment cost based on the proposed implementation of HAART expansion based on the 2008 IAS-USA guidelines for the 50% and 75% scenarios (Figure 3). We estimated that after 20 years, the 50% scenario will yield high returns, and this period is shortened by 5 years if expansion is done based on the 75% scenario.

## Discussion

Our results demonstrated that HAART expansion to those in need, based on the 2008 IAS-USA guidelines in British Columbia, will be expected to result in an immediate decrease in HIV/AIDS morbidity and mortality and a decrease in HIV incidence. Indeed, we showed that in 5 years, we could avert at least 1360 infections, avert loosing 4155 years due to disability and mortality and approximately CAD$ 21 million in averted costs by just increasing HAART coverage to 50% of eligible individuals. The benefits were even bigger is expansion is done for the 75% scenario, instead. The averted lifelong costs associated with the initiative were also significant, and the timing for the return of investment was directly and strongly associated with how many more patients will be coved by HAART expansion. However, despite of costs associated with this initiative, we have shown that there will be huge gains in healthy years and in decreasing premature mortality of those infected with HIV in the short and long terms.

However, there are tremendous challenges associated with this initiative, given that a substantial number of individuals eligible for treatment will come from the pool of those marginalized and not engaged in care. The HAART expansion based on the 2008 IAS-USA guidelines will therefore require the development of a sustained and intensified strategy to identify HIV infected individuals and those at risk, helping them to stabilize their lives and facilitate their sustained engagement in appropriate health care and support, all of this within a voluntary and fully consented framework. These individuals are usually suffering from mental illnesses, addiction and poverty, thus complicating their access to proper health care, as it is the case of a large number of Aboriginal peoples and injection drug users. Of note, the estimated 25%–30% of HIV-infected individuals who are unaware of their HIV status represents a major unresolved challenge. Further focused efforts are underway to conduct feasibility studies to massively test individuals to address this deficit, and provide valuable data for other HAART expansion programs [40]. Additionally, it is worthy mentioned that this model was based on the epidemic in BC, which is concentrated among MSMs and IDUs, which is the opposite of other parts of the world which have generalized epidemics, and therefore the results of this model should be taken cautiously. Another model limitation, which directly affects the HAART expansion initiative, is the fact that we did not account for loss of follow-up. There is work under way to estimate in our program what is the percentage of individuals lost to follow-up, how much such percentage might change over time, and its impact on population-level viremia. Finally, the proposed HAART expansion based on the 2008 IAS-USA guidelines should take place within a concerted effort to strengthen other evidence based preventive interventions, including education, risk reduction and harm reduction strategies [41]–[43].

To date, HAART has dramatically decreased morbidity and mortality among HIV infected individuals around the world. HAART expansion based on the 2008 IAS-USA guidelines has a tremendous potential to further improve health outcomes among HIV infected individuals in BC and in the rest of the world and, furthermore, to substantially change the course of the HIV epidemic by decreasing rates of HIV transmission, tuberculosis, malaria and other infectious diseases and therefore curbing the growth of the epidemic [41]. However, all these benefits come with a high price. The best tolerated and efficient antiretroviral drugs are still quite expensive and unaffordable in parts of the world, and sometimes have unwanted side effects such as antiretroviral resistance. Recently there was a mathematical model that showed that drug resistance can pose a threat to expansion HAART initiatives [44]. Yet, our group has empirically shown that population-level drug resistance has been declining in BC since 1996, due to an increasing number of individuals achieving virologic suppression in our program, and therefore we believe that drug resistance in settings like ours might not pose any threat to an increased number of individuals receiving HIV care [45]. In addition, delivering proper care to all of those who meet medical eligibility criteria will require substantial efforts from public and private sectors, and the urgent address of underlying systemic challenges such as stigma, discrimination, inadequate healthcare infrastructure, lack of education, poor nutrition, homelessness, insufficient mental health services, lack of addiction management services, including drug substitution and harm reduction.

In summary, our results suggest that the implementation of the revised IAS-USA guidelines for use of HAART in adults can have a dramatic impact not just on the health of HIV infected individuals but also on the spread of HIV. These results provide powerful additional evidence regarding the need to ensure that individuals in medical need of HAART can have access to this lifesaving intervention for their own benefit, as well as, a way to curb the growth of the epidemic. While implementing the revised IAS-USA guidelines for use of HAART in adults may be associated with significant challenges, including up front costs, our analysis demonstrates that this is highly cost-effective and cost-averting within a decade.

## Supporting Information

Table S1 Definition and value for the parameters in the transmission model.
(0.09 MB DOC)

Figure S1 Distribution of HIV infected individuals in British Columbia engaged and not engaged in care. Groups are defined as follows: (1) individuals currently receiving HIV treatment and care (N = 4379); (2) individuals who are currently engaged in care, who now meet the eligibility to start HAART under the new guidelines (N = 1602); (3) same as group 2, but for personal reasons, despite being well adjusted in society and engaged in medical care, are reluctant to initiate treatment (N = 600); (4) individuals who are currently engaged in care, but yet do not meet the eligibility to start HAART under the new guidelines (N = 798); (5) represents the current deficit of individuals (not engaged in care) who should be on treatment under the previous guidelines because their CD4 cell count is <200 cells/mm3; (6) and (7) individuals not engaged in care, who now meet the eligibility to start HAART under the new guidelines because their CD4 cell count is <350 cells/mm3 (N = 1036) or because they meet at least one of the other criteria for therapy initiation (N = 1476); (8) individuals who are not engaged in care, and do not meet the eligibility to start HAART under the new guidelines (N = 342).
(0.45 MB TIF)
