# Supplementary material for: Expanding HAART Treatment to All Currently Eligible Individuals under the 2008 IAS-USA Guidelines in British Columbia, Canada
Source: PLoS One. 2010 Jun 7;5(6):e10991. doi: 10.1371/journal.pone.0010991 (PMC2881871; doi:10.1371/journal.pone.0010991)
Supplement: Table S1 — Definition and value for the parameters in the transmission model. (0.09 MB DOC) [file pone.0010991.s001.doc]

| **Parameter** | **Definition** | **Values** |
| --- | --- | --- |
| $\pi$ | Recruitment rate for the HIV-negative population at high risk of infection. | 1318 individuals per year. |
| $\gamma$ | Rate at which an individual infected and waiting to meet eligibility to start treatment, meets eligibility and starts treatment. | $\frac{1}{12}\times\left(\text{waiting time}\right)^{-1}$, for waiting time being 1.5 years. |
| $\phi$ | Proportion of individuals infected and waiting to meet eligibility to start treatment, meets eligibility and do not start treatment. | Based on past enrolment statistics of the Drug Treatment Program of the British Columbia Centre for Excellence in HIV/AIDS. |
| $\varphi\left(t\right)$ | Proportion of individuals infected eligible to start treatment, do start treatment. | Based on the British Columbia Ministry of Health requirements: 20% per year during first 5 years, and 10% the years thereafter. |
| $\lambda\left(t\right)$ | Force of infection. | $\begin{matrix} \lambda\left(t\right)=\frac{\left(\beta_{1}\times p_{1}^{T}+\beta_{2}\times p_{2}^{T}+\beta_{3}\times p_{3}^{T}+\beta_{4}\times p_{4}^{T}\right)\times T\left(t\right)}{N\left(t\right)}\text{ }+ \\ \text{ }\frac{\left(\beta_{1}\times p_{1}^{U}+\beta_{2}\times p_{2}^{U}+\beta_{3}\times p_{3}^{U}+\beta_{4}\times p_{4}^{U}\right)\times(W\left(t\right)+U\left(t\right))}{N\left(t\right)} \end{matrix}$ |
| $\beta_{1}$,$\beta_{2}$,  $\beta_{3}$,$\beta_{4}$ | Transmission probabilities for each of the HIV-1 RNA viral load strata (<3 log_10_ copies/mL, ≥3 and <4 log_10_ copies/mL, ≥4 log_10_ copies/mL) and Late Stage (i.e. CD4 cell count <200 cells/mm^3^). | 1. Based on Gray RH, Wawer MJ, Brookmeyer R, Sewankambo NK, Serwadda D, Wabwire-Mangen F, et al ; Rakai Project Team. Probability of HIV-1 transmission per coital act in monogamous, heterosexual, HIV-1-discordant couples in Rakai, Uganda. Lancet 2001;357:1149-53.  2. Wawer MJ, Gray RH, Sewankambo NK, Serwadda D, Li X, Laeyendecker O, et al. Rates of HIV-1 transmission per coital act, by stage of HIV-1 infection, in Rakai, Uganda. J Infect Dis 2005;191:1403-9. |
| $p_{1}^{T}\left(t\right)$,$p_{2}^{T}\left(t\right)$,  $p_{3}^{T}\left(t\right)$,$p_{4}^{T}\left(t\right)$ | Time dependent probability of being in each of the HIV-1 RNA viral load strata (<3 log_10_ copies/mL, ≥3 and <4 log_10_ copies/mL, ≥4 log_10_ copies/mL) and Late Stage (i.e. CD4 cell count <200 cells/mm^3^) for those on treatment. | Estimated via partial proportion odds model at each time period (6 mo, 12mo, 18mo, etc.), in which the outcome is HIV-1 RNA viral load strata, and adjusted for time period (6 mo, 12mo, 18mo, etc.), age gender, and history of injection drug use (yes/no), time-dependent adherence, baseline HIV-1 RNA viral load, time-dependent CD4 cell count. The analyses were performed using SAS software (version 9.1.3, service pack 3). |
| $p_{1}^{U}\left(t\right)$,$p_{2}^{U}\left(t\right)$,  $p_{3}^{U}\left(t\right)$,$p_{4}^{U}\left(t\right)$ | Time dependent probability of being in each of the HIV-1 RNA viral load strata (<3 log_10_ copies/mL, ≥3 and <4 log_10_ copies/mL, ≥4 log_10_ copies/mL) and Late Stage (i.e. CD4 cell count <200 cells/mm^3^) for those not on treatment. | Based on Lima VD, Fink V, Yip B, Hogg RS, Harrigan PR, Montaner JS. Association between HIV-1 RNA level and CD4 cell count among untreated HIV-infected individuals. Am J Public Health 2009;99 (Suppl 1):S193-6. |
| $\mu{}_{1}$,$\mu{}_{2}$,  $\mu_{3}$,$\mu_{4}$ | All cause (Non AIDS and AIDS-related) mortality rates for HIV-positive individuals and all cause mortality for HIV-negative individuals. | HIV-negative individuals: obtained via the British Columbia Vital Statistics Agency in 2006 – 0.006966 per year  HIV-positive individuals on treatment: obtained via of the Drug Treatment Program of the British Columbia Centre for Excellence in HIV/AIDS in 2006 – 0.0064185 per year  HIV-positive individuals not on treatment: assumed to be twice of the rate of those on treatment.  Survival rates were estimated for those on HAART and not on HAART. We built an explanatory model using Cox-proportional hazard regression, and the assumption of proportional hazards was examined graphically. The analyses were performed using SAS software (version 9.1.3, service pack 3). |
| $S\left(t_{0}\right)$,$W\left(t_{0}\right)$ $T\left(t_{0}\right)$,$\text{ }U\left(t_{0}\right)$ | Initial values for each of the compartments. | Based on the numbers in Figure 2. |
