# Supplementary figures and images for: Expanding HAART Treatment to All Currently Eligible Individuals under the 2008 IAS-USA Guidelines in British Columbia, Canada
Source: PLoS One. 2010 Jun 7;5(6):e10991. doi: 10.1371/journal.pone.0010991 (PMC2881871; doi:10.1371/journal.pone.0010991)

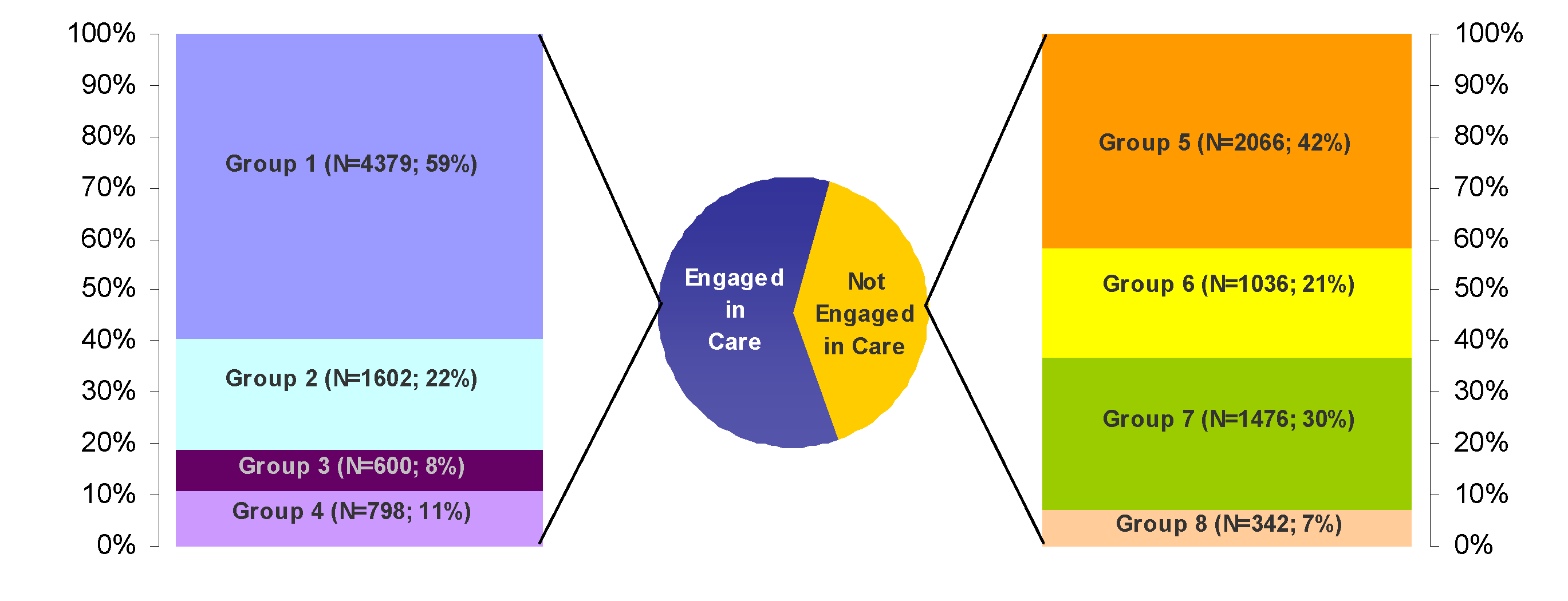

Supplement: Figure S1 — Distribution of HIV infected individuals in British Columbia engaged and not engaged in care. Groups are defined as follows: (1) individuals currently receiving HIV treatment and care (N = 4379); (2) individuals who are currently engaged in care, who now meet the eligibility to start HAART under the new guidelines (N = 1602); (3) same as group 2, but for personal reasons, despite being well adjusted in society and engaged in medical care, are reluctant to initiate treatment (N = 600); (4) individuals who are currently engaged in care, but yet do not meet the eligibility to start HAART under the new guidelines (N = 798); (5) represents the current deficit of individuals (not engaged in care) who should be on treatment under the previous guidelines because their CD4 cell count is <200 cells/mm3; (6) and (7) individuals not engaged in care, who now meet the eligibility to start HAART under the new guidelines because their CD4 cell count is <350 cells/mm3 (N = 1036) or because they meet at least one of the other criteria for therapy initiation (N = 1476); (8) individuals who are not engaged in care, and do not meet the eligibility to start HAART under the new guidelines (N = 342). (0.45 MB TIF) [file pone.0010991.s002.tif]
